# Acute kidney injury is an independent risk factor for pediatric intensive care unit mortality, longer length of stay and prolonged mechanical ventilation in critically ill children: a two-center retrospective cohort study

**DOI:** 10.1186/cc10269

**Published:** 2011-06-10

**Authors:** Omar Alkandari, K Allen Eddington, Ayaz Hyder, France Gauvin, Thierry Ducruet, Ronald Gottesman, Véronique Phan, Michael Zappitelli

**Affiliations:** 1 Division of Nephrology, Department of Pediatrics, McGill University Health Centre, Montreal Children's Hospital, 2300 Tupper, Room E-213, Montreal, QC, H3H 1P3, Canada; 2 Division of Critical Care Medicine, Department of Pediatrics, Université de Montréal, Centre Hospitalier Universitaire Sainte-Justine, 3175, Chemin de la Côte-Sainte-Catherine, Bureau 3434, Montreal, QC, H3T 1C5, Canada; 3 Centre Hospitalier Universitaire Sainte-Justine, Groupe de recherche clinique en soins intensifs pédiatriques (GRC-SIP), Centre de Recherche Bureau A-551, Chemin de la Côte-Sainte-Catherine, Bureau Montreal, QC, H3T 1C5, Canada; 4 Division of Critical Care Medicine, Department of Pediatrics, McGill University Health Centre, Montreal Children's Hospital, 2300 Tupper, C-808, Montreal, QC, H3H 1P3, Canada; 5 Division of Nephrology, Department of Pediatrics, Université de Montréal, Centre Hospitaler Universitaire Sainte-Justine, Chemin de la Côte-Sainte-Catherine, Bureau 2403, Montreal, QC, H3T 1C5, Canada

## Abstract

**Introduction:**

In adults, small (< 50%) serum creatinine (SCr) increases predict mortality. It is unclear whether different baseline serum creatinine (bSCr) estimation methods affect findings of acute kidney injury (AKI)-outcome associations. We characterized pediatric AKI, evaluated the effect of bSCr estimation approaches on AKI-outcome associations and evaluated the use of small SCr increases to predict AKI development.

**Methods:**

We conducted a retrospective cohort database study of children (excluding postoperative cardiac or renal transplant patients) admitted to two pediatric intensive care units (PICUs) for at least one night in Montreal, QC, Canada. The AKI definition was based on the Acute Kidney Injury Network staging system, excluding the requirement of SCr increase within 48 hours, which was impossible to evaluate on the basis of our data set. We estimated bSCr two ways: (1) the lowest SCr level in the three months before admission or the average age- and gender-based norms (the standard method) or (2) by using average norms in all patients. Outcomes were PICU mortality and length of stay as well as required mechanical ventilation. We used multiple logistic regression analysis to evaluate AKI risk factors and the association between AKI and mortality. We used multiple linear regression analysis to evaluate the effect of AKI on other outcomes. We calculated diagnostic characteristics for early SCr increase (< 50%) to predict AKI development.

**Results:**

Of 2,106 admissions (mean age ± SD = 5.0 ± 5.5 years; 47% female), 377 patients (17.9%) developed AKI (using the standard bSCr method) during PICU admission. Higher Pediatric Risk of Mortality score, required mechanical ventilation, documented infection and having a bSCr measurement were independent predictors of AKI development. AKI was associated with increased mortality (adjusted odds ratio (OR) = 3.7, 95% confidence interval (95% CI) = 2.1 to 6.4, using the standard bSCr method; OR = 4.5, 95% CI = 2.6 to 7.9, using normative bSCr values in all patients). AKI was independently associated with longer PICU stay and required mechanical ventilation. In children with no admission AKI, the initial percentage SCr increase predicted AKI development (area under the curve = 0.67, 95% CI = 0.60 to 0.74).

**Conclusions:**

AKI is associated with increased mortality and morbidity in critically ill children, regardless of the bSCr used. Paying attention to small early SCr increases may contribute to early AKI diagnosis in conjunction with other new AKI biomarkers.

## Introduction

Acute kidney injury (AKI) is well known to be associated with longer hospital length of stay (LOS), morbidity and mortality in adults [1,2]. In recent years, it has become apparent that even very small increases in serum creatinine (SCr) levels in hospitalized adults and in children undergoing cardiac surgery are associated with poor hospital outcomes [3,4]. Most previous research in pediatric AKI was focused on patients requiring acute dialysis. Recently derived AKI definitions now allow us to comprehensively evaluate risk factors for and outcomes of AKI [5,6], which is a necessary first step to perform prior to initiating clinical trials to reduce AKI incidence and improve AKI outcomes.

Single-center studies have been performed in select pediatric populations using one of two similar standardized AKI definitions: the pediatric Risk, Injury, Failure, Loss, End-Stage Renal Disease criteria (pRIFLE) [5] or the Acute Kidney Injury Network (AKIN) staging system [6]. These studies have reported variable AKI incidence in critically ill children, largely due to variations in illness severity and in inclusion/exclusion criteria of study populations. However, all of these studies have found that AKI is associated with poor outcomes (such as longer LOS or mortality) [4,7-9]. Schneider *et al*. [8] recently published the largest single-center database study of all children admitted to the critical care unit. They reported an overall AKI rate of 10% and found that AKI was independently associated with increased mortality and hospital LOS. This study was extremely important because of its large study population and because of its detailed description of the timing of AKI development.

There is still much to learn about AKI epidemiology and disease description in children. First, all previous studies were single-center studies; therefore, validation of findings from previous studies using large cohorts is necessary. Second, it is still unclear to what extent the use of differing definitions for baseline kidney function affects AKI disease descriptions and AKI associations with outcomes when using large database studies. This is particularly relevant, since baseline kidney function is often unknown in children and the definition of AKI is structured upon examining SCr increases relative to baseline SCr (bSCr) values.

The following were the aims of the current study: (1) to determine the incidence of AKI according to standard AKI definitions and to elucidate AKI risk factors in two tertiary healthcare center pediatric intensive care units (PICUs) for the purpose of validating and comparing findings from other recent studies, (2) to determine to what extent the use of different ways of estimating bSCr levels affects inferences regarding the association between AKI and outcomes and (3) to evaluate how early small increases in SCr might predict the future development of more significant AKI.

## Materials and methods

### Design, setting and patient selection

We conducted a retrospective database cohort study at two tertiary pediatric healthcare centers in Montreal, QC, Canada: the Montreal Children's Hospital (MCH) and the Centre Hospitalier Universitaire Ste-Justine (CHUSJ). PICU clinical databases available at each institution were combined. Inclusion criteria were admission to the PICU for at least 12 hours and at least one night and age 18 years old or younger at the time of PICU admission. Exclusion criteria were the primary reason for PICU admission being postoperative cardiac surgery or renal transplantation. Repeat PICU admissions were not excluded. Institutional research ethics board approval was obtained from both centers to combine both databases, and the requirement for patient consent was waived. The analysis and reporting of this study were performed with guidance from the Strengthening the Reporting of Observational Studies in Epidemiology statement checklist [10].

### Description of clinical databases

#### Montreal children's hospital database

The Pediatric Intensive Care Evaluations (PICUEs) version 3.2.3 database is an institutional clinical database which was maintained between 2003 and 2007 at MCH for quality assurance monitoring. Data were collected prospectively by PICU staff. A nurse practitioner performed detailed chart reviews for missing data or for inconsistencies regarding admissions occurring between 1 September 2004 and 30 September 2007. The PICUEs database includes multiple demographic, clinical and outcome variables, which are required for calculation of the Pediatric Risk of Mortality (PRISM) score [11], but no data regarding daily SCr variables.

#### Centre hospitalier universitaire ste-justine database

The CHUSJ database was derived from a previous prospective observational study (between 10 January 2000 and 9 January 2001) of AKI in children admitted to the PICU conducted prior to the publication of the pRIFLE definition of AKI [12]. All consecutive PICU admissions during this period were included in the original study database unless they met the following exclusion criteria: < 3 days of age or < 40 weeks of gestation, > 18 years of age, pregnancy or postpartum admission, admission for renal transplantation, brain death at entry into the PICU, expected PICU LOS < 24 hours, *a priori *decision to withhold or withdraw treatment and end-stage renal failure (62 children met the exclusion criteria). Demographic, clinical and outcome variables as well as variables required for calculation of PRISM scores were collected prospectively by PICU staff.

#### Variables common to both databases extracted for this study

Investigators examined case report forms for each database to decide which variables could reliably be combined from both databases. The following variables were extracted from each database because they were collected using similar criteria and because they were relevant risk factors or outcomes of AKI: age at PICU admission, gender, PRISM score, requirement for mechanical ventilation during PICU admission, admission diagnosis of trauma, documented infection during PICU admission, admission diagnosis of postoperative care (noncardiac), PICU LOS and duration of mechanical ventilation, and PICU mortality. Hospital LOS and hospital mortality were not readily available from the CHUSJ database; therefore, these outcomes were not evaluated. The Pediatric Logistic Organ Dysfunction scores [13] were available in both databases, but several scoring variables were missing in approximately 150 patients. Moreover, this measure includes a renal score, which could lead to colinearity problems with AKI; therefore, only the PRISM score was evaluated.

### Hospital laboratory database collection of serum creatinine

Neither database contained SCr data. The MCH investigators had previously collected all SCr values from all patients included in the MCH PICUEs database for a previous study [4]. The CHUSJ investigators collected SCr values specifically for this study.

The primary method of determining bSCr was to record the lowest SCr level within the three months prior to PICU admission. When bSCr was unavailable, we used the average SCr norms for age and gender specific to each institution's biochemistry laboratory [14]. Hereinafter we refer to this method of determining baseline SCr as the "standard bSCr method." We were careful to use each laboratory's SCr norms corresponding to the respective period of study (for example, SCr norms of 2000 and 2001 at CHUSJ).

We recorded the highest SCr level obtained during PICU admission for calculation of maximal AKI status (peak SCr increase relative to bSCr), and we also recorded the last PICU SCr level measured. From the MCH database, we additionally had all SCr values obtained during PICU admission. To determine the extent to which SCr levels returned to bSCr toward the end of PICU admission, we calculated the percentage differences and the raw differences (in micromoles per liter) between last PICU SCr level and bSCr.

### AKI definition

The pRIFLE pediatric AKI definition classifies AKI based on acute reductions in estimated creatinine clearance [5]; however, we did not have height data available for most patients, making it impossible to calculate estimated creatinine clearance [15]. We therefore defined AKI based on the AKIN staging system [6]. The AKIN staging system definition grades AKI as AKIN stage 1 if SCr increases to ≥ 1.5 × bSCr level or by ≥ 26 μmol/L, as AKIN stage 2 if SCr level doubles and as AKIN stage 3 if SCr triples or if the patient requires dialysis. Of note, the AKIN staging system stipulates that SCr should increase within 48 hours for AKI to be defined. We were unable to utilize this criterion, since not all patients had SCr measured on consecutive days. Therefore, any patient who fulfilled the AKIN staging criteria for SCr increase at any time was classified as having AKI. There were no urine output data available for analysis. However, previous studies have suggested that the inclusion or exclusion of urine output data did not significantly alter AKIN stage designation [5,16].

### Comparison of different methods for estimating bSCr

We calculated AKI status for each patient by using two different ways of estimating bSCr. The first method, as described above, defined bSCr as the lowest SCr level measured during the three months before PICU admission or using average age- and gender-based norms when there were no prior SCr data available. Since this is the standard way of estimating bSCr, hereinafter we refer to AKI defined in this way as AKIN_standard bSCr_. (2) To simulate the situation in which no bSCr values are available for any patients (as in the setting of a database study), we used average age- and gender-based normative SCr values to estimate bSCr for all patients to determine AKI status. For simplicity, we refer to this method of AKI classification as AKIN_all norms bSCr _to denote that normative bSCr values were used for all patients.

### Outcomes

Clinical outcomes were PICU mortality, PICU LOS and duration of mechanical ventilation.

### Statistical analysis

Continuous variables were expressed as means ± standard deviations (SDs) or as medians and categorical variables expressed as percentages. Paired *t*-tests were used to compare continuous variables, and Pearson's χ^2 ^test was used to compare categorical variables.

Backward stepwise multiple logistic regression (*P *value exit criteria = 0.2) was performed to evaluate independent risk factors for developing AKI, including the following variables: age (less than one year old or one year old or older), gender, study site, PRISM score (expressed in tertile categories), need for mechanical ventilation (yes or no), admission diagnosis of trauma (yes or no), documented infection (yes or no), postoperative PICU admission diagnosis (yes or no) and availability of bSCr (yes or no). Age, gender and study site data were forced into the model.

Stepwise multiple logistic regression analysis was used to evaluate the independent effect of AKI on PICU mortality. We first evaluated a simpler regression model controlling for age, gender and study site (model 1). We then performed backward stepwise logistic regression analysis, forcing AKI, age, gender and study site into the model and evaluating the effects of PRISM score, need for mechanical ventilation, postoperative diagnosis, documented infection, availability of bSCr and trauma diagnosis (model 2), and using an exit *P *value criterion of 0.2. Evaluation of interaction terms of AKI and other variables was attempted; however, because of the low number of outcomes, regression models were unstable.

We used multiple linear regression analysis to evaluate the effect of AKI on PICU LOS and on duration of mechanical ventilation independent of the effect of other variables described above. In cases of non-normal distribution, PICU LOS and duration of mechanical ventilation were natural logarithm-transformed for these analyses.

All analyses were performed using both bSCr methods to define AKI (using the standard bSCr method or AKIN_standard bSCr _and by estimating bSCr using normative values in all patients or AKIN_all norms bSCr_). Moreover, all analyses were performed expressing AKI as any AKI occurring during admission (AKIN stage 1 or worse) and then again expressing AKI as stage 2 or worse to evaluate the effect of more severe AKI on outcomes.

Because we had SCr values available from all PICU admission days in the MCH database, we performed the following secondary analyses utilizing only the MCH database. We calculated and described the first day of PICU admission on which patients developed AKI. In patients who did not yet have AKI on the first day of PICU admission and in whom SCr measurement was available on that day, we determined the ability of percentage SCr increase on admission day 1 to predict future development of AKI by constructing a receiver operating characteristic curve, calculating area under the curve (AUC) and evaluating sensitivity and specificity of different percentage SCr increase cutoffs. The purpose of that analysis was to determine whether, in a patient who does not yet have AKI, the extent of SCr increase (even if small) can predict whether that patient will progress toward the development of AKI. While there was no formal sample size analysis performed prior to initiating the study, our goal was to have enough mortality data in our database to be able to control for six to eight variables in multivariate analyses. Given our fixed sample size and the ability to achieve this goal, we did not supplement our databases with more patients by reviewing medical charts retrospectively. STATA SE software version 10 (StataCorp, College Station, TX, USA) was used to perform all statistical analyses.

## Results

### Study population

**Figure 1:**
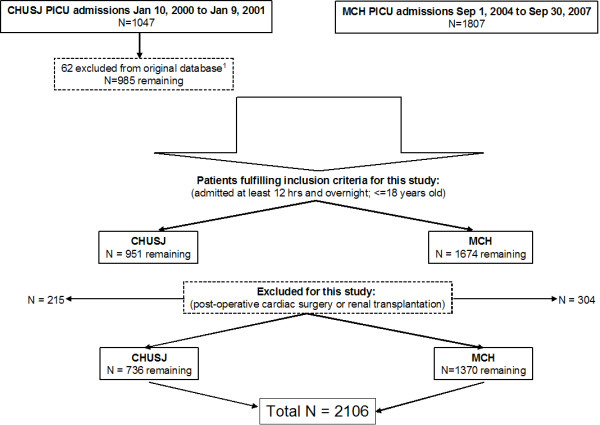
**Study inclusion and exclusion criteria flowchart**. This flowchart illustrates the inclusion and exclusion criteria used to screen patients from both study centers to derive the final study population. ^1^Centre Hospitalier Universitaire Ste-Justine (CHUSJ) original exclusion criteria were < 3 days of age or < 40 weeks of gestation, > 18 years of age, pregnancy or postpartum admission, admission for renal transplantation, brain death at entry into the pediatric intensive care unit (PICU), expected PICU stay < 24 hours, *a priori *decision to withhold or withdraw treatment and end-stage renal failure. hrs = hours; Jan = January; MCH = Montreal Children's Hospital; Sep = September.

Figure 1 is the inclusion and exclusion flowchart, which shows that 2,106 patients were available for analysis, composed of 35% from the CHUSJ database and 65% from the MCH database. The mean ± SD (median) age of the cohort was 5.8 ± 5.7 (3.6) years, and the mean ± SD (median) PRISM score of the cohort was 5.4 ± 5.1 (4). Among the total patient group, 918 (44%) of patients were female, 973 (46%) needed mechanical ventilation, 726 (35%) were postoperative patients (noncardiac), 147 (7%) had a primary diagnosis of trauma and 106 (5%) had a documented infection. Thirty (1.4%) of all patients received renal replacement therapy (same proportion at both centers). Comparison of the MCH and CHUSJ databases revealed that there were no statistically significant differences regarding age (mean 5.9 years versus 5.7 years; *P *= 0.6), proportion of female patients (43% versus 45%; *P *= 0.4), proportion of postoperative patients (33% versus 37%; *P *= 0.1), proportion of patients with a trauma diagnosis (both 7%) or proportion of patients with a documented infection (both 5%). However, patients from the MCH database had a slightly lower mean PRISM score (5.2 versus 5.7; *P *= 0.05) and a higher proportion of patients who required mechanical ventilation (52% versus 36%; *P *< 0.01). Given the discrepancy in ventilation status, we reviewed data from another study at CHUSJ during a similar time period (2000 and 2001) and confirmed the accuracy of the proportion of patients requiring ventilation.

### Incidence of acute kidney injury

Of all patients, 975 (46%) had bSCr data available from the previous three months (46% from the MCH database and 45% from the CHUSJ database; *P *= 0.6). Using the standard method of determining bSCr, 377 (17.9%) of patients developed AKI (AKIN_standard bSCr_) during PICU admission, with 206 patients (9.8% of the total sample) diagnosed with stage 1 AKI, 91 patients (4.3%) diagnosed with stage 2 AKI and 80 patients (3.8%) diagnosed with stage 3 AKI. When using the average age- and gender-based SCr normative values to estimated bSCr in all subjects (AKIN_all norms bSCr_), 15.1% of patients were classified as having AKI, comprising 171 patients (8.1%) with stage 1 AKI, 72 patients (3.4%) with stage 2 AKI and 76 patients (3.6%) with stage 3 AKI.

### Agreement in acute kidney injury ascertainment comparing the two baseline serum creatinine measurement methods

On the basis of examination of the whole sample, there was 91% agreement between the two AKI definition methods (AKIN_standard bSCr _versus AKIN_all norms bSCr_: κ = 0.70, *P *< 0.001 for AKIN staging AKI severity). When only patients who had bSCr data available (*n *= 975) were examined, there was 81% agreement in AKIN staging (κ = 0.42, *P *< 0.001). Only 3% of patients classified as having no AKI using the standard bSCr method (AKIN_standard bSCr_) were found to have AKI when normative bSCr values were used in all patients (AKIN_all norms bSCr_), whereas 6% of patients classified as having no AKI when bSCr was estimated on the basis of normative values in all patients (AKIN_all norms bSCr_) were found to have AKI when bSCr was estimated on the basis of the standard method (AKIN_standard bSCr_).

### AKI risk factors

The evaluated AKI risk factors are shown in Table 1, where AKI and non-AKI groups are compared. In multivariate analysis, independent statistically significant risk factors for developing AKI were higher PRISM score, need for mechanical ventilation, documented infection, bSCr measured and not being a postoperative patient (odds ratios shown in Table 1).

**Table 1:** Patient characteristics by AKI status and variable associations with AKI^a,b^

	Mean (± SD), median or *n *(%)^e^			
Characteristics (*N *= 2,106)	AKI (*n *= 377)	Non-AKI (*n *= 1,729)	*P *value^c^	AKI risk factors adjusted ORs (95% CI)^d^
AKI risk factors				
Age, years	5.0 (5.5), 2.2	6.0 (5.7), 4.0	0.002	0.99 (0.97 to 1.01)
PRISM score^f^	7.9 (6.3), 7	4.8 (4.6), 4	< 0.001	1.10 (1.08 to 1.13)^g^
Centre Hospitalier Universitaire Ste-Justine	116 (30.8%)	620 (35.9%)	0.06	0.79 (0.61 to 1.02)
Female gender	161(42.7%)	757 (43.8%)	0.7	1.00 (0.79 to 1.27)
Mechanically ventilated	226 (60.0%)	747 (43.2%)	< 0.001	1.52 (1.18 to 1.94)^g^
Measured bSCr	218 (57.8%)	757 (43.8%)	< 0.001	2.22 (1.72 to 2.86)^g^
Postoperative (noncardiac)	102 (27.1%)	624 (36.1%)	0.001	0.68 (0.51 to 0.90)^g^
Admission for trauma	15 (4.0%)	132 (7.6%)	0.01	0.59 (0.33 to 1.07)
Documented infection	39 (10.3%)	67 (3.9%)	< 0.001	1.92 (1.23 to 2.99)^g^
Outcomes				
Length of mechanical ventilation, days	5.4 (9.7), 1	2.2 (8.6), 0	< 0.001	Not applicable
PICU length of stay, days	9.7 (21.7), 3.1	4.6 (16.2), 2	< 0.001	Not applicable
PICU mortality	39 (10.3%)	30 (1.7%)	< 0.001	Not applicable

^a^AKI = acute kidney injury; bSCr = baseline serum creatinine; I = confidence interval; OR = odds ratio; PICU = pediatric intensive care unit; PRISM = Pediatric Risk of Mortality; SD = standard deviation;

^b^AKI was defined according to the traditional Acute Kidney Injury Network staging system by using the lowest SCr level in the previous three months or age- and gender-based normative values as bSCr. Patients with no SCr data available were assumed not to have developed AKI; ^c^*P *values are based on the univariate comparison test performed between AKI and non-AKI groups; ^d^adjusted ORs were calculated based on multiple logistic regression analysis to evaluate independent risk factors for AKI; ^e^*n *(%) data represent the column percentage (for example, proportion of AKI patients who were female and proportion of AKI patients who were treated at Centre Hospitalier Universitaire Ste-Justine); ^f^only 2,085 patients had PRISM scores available; ^g^statistically significant (*P *< 0.05) ORs represent independent AKI risk factors.

### Last pediatric intensive care unit serum creatinine measurement

**Figure 2:**
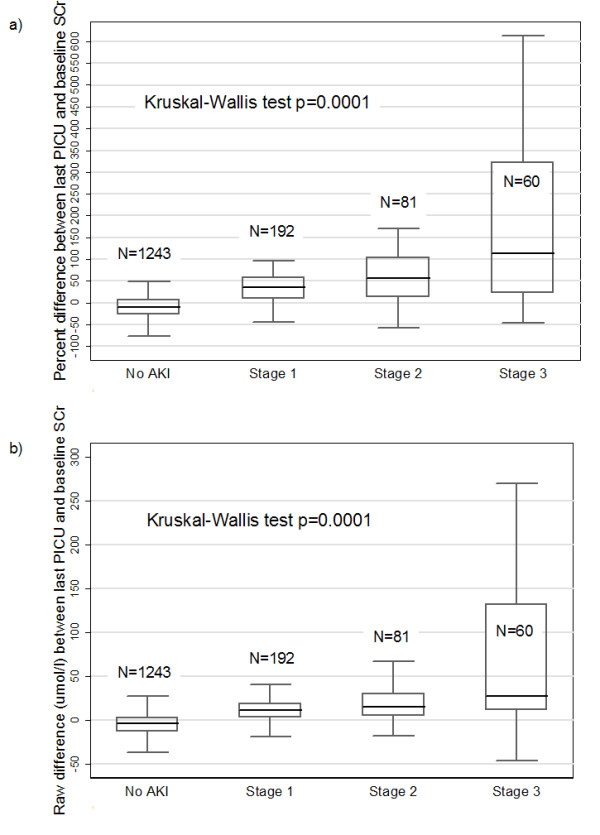
**Difference between last PICU SCr and baseline SCr based on AKI status**. Boxplots illustrating **(a) **percentage differences between last pediatric intensive care unit (PICU) serum creatinine (SCr) and baseline SCr (bSCr) and **(b) **raw differences (in μmol/L) between last PICU SCr and bSCr levels in patients who survived through PICU discharge and in whom at least one SCr measurement was performed. Boxplots are presented according to Acute Kidney Injury Network (AKIN) staging system status from no acute kidney injury (AKI) (far left) to AKIN stage 3 (far right). A nonparametric Kruskal-Wallis test was used to evaluate significant differences across the four groups.

Among patients who had at least one SCr measurement and survived to PICU discharge (*n *= 1,576), 322 patients (20.4%) had a last PICU SCr value ≥ 25% above bSCr. Among the 789 of these patients who had a bSCr level available within three months before PICU admission, the proportion of patients whose last PICU SCr value was ≥ 25% above bSCr was 25%. Among patients with a normative value-based bSCr, 16% had a last PICU SCr value at least 25% above bSCr. Figure 2a demonstrates that the percentage increase of the last PICU SCr level from the bSCr increased in a graded and statistically significant fashion (*P *= 0.0001, Kruskal-Wallis test) from the no AKI category to the AKIN Stage 3 classification at both study centers. Figure 2b demonstrates a similar finding on the basis of using the raw differences (in micromoles per liter) between the last PICU SCr and bSCr values.

### Timing of first acute kidney injury

**Figure 3:**
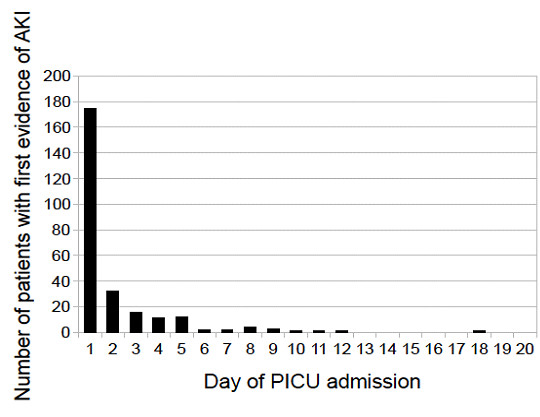
**Day of PICU admission when AKI first occurred at the Montreal Children's Hospital center**. Histograms of patients with acute kidney injury (AKI) depicting the number of patients who first developed AKI on each day of pediatric intensive care unit (PICU) admission. Data are representative of only the Montreal Children's Hospital center.

In MCH-specific analyses, 1,077 patients had a SCr measurement performed on day 1 or day 2 of PICU admission. Among only those patients, 207 (19.2%) had AKI on day 1 or day 2 of PICU admission. Figure 3 shows that, overall, almost all AKIs occurred within the first two or three days after PICU admission.

### Acute kidney injury as a risk factor of pediatric intensive care unit mortality

There were 69 PICU deaths, with mortality being approximately six times higher in patients with AKI (Table 1). In stepwise multiple logistic regressions analyses, AKI was a persistent risk factor for mortality, independent of the effects of other variables and regardless of how bSCr was defined (see Tables 2 and 3). In addition, the association of stage 2 AKI or worse and mortality was always slightly stronger than the presence of any AKI, again, regardless of the bSCr measurement method used (Table 3). Other persistent independent risk factors of mortality were PRISM score (highest tertile), requirement for ventilation and documented infection. Patients' postoperative status was a persistent protective factor for mortality (see Tables 2 and 3).

**Table 2:** Association of any AKI with PICU mortality using two different approaches for determination of bSCr levels^a^

	Standard bSCr determination (AKIN_standard bSCr_)^b^		Normative values for bSCr in all subjects (AKIN_all norms bSCr_)^b^	
Characteristics	Model 1^c^	Model 2^c^	Model 3^c^	Model 4^c^
AKI	6.5 (4.0 to 10.7)^d^	3.7 (2.1 to 6.4)^d^	8.4 (5.1 to 14.0)^d^	4.5 (2.6 to 7.9)^d^
Age under one year	1.6 (1.0 to 2.7)	1.0 (0.6 to 1.7)	1.8 (1.1 to 3.0)^d^	1.0 (0.6 to 1.7)
Female	0.6 (0.4 to 1.1)	0.7 (0.4 to 1.5)	0.6 (0.4 to 1.1)	0.7 (0.4 to 1.2)
CHUSJ site	1.5 (0.9 to 2.5)	1.9 (1.1 to 3.3)^d^	1.8 (1.1 to 3.0)^d^	2.3 (1.3 to 4.0)^d^
PRISM score, tertiles				
0 to 3 reference group	-	1.0 (reference)	-	1.0 (reference)
4 to 6	-	0.5 (0.2 to 1.5)	-	0.5 (0.2 to 1.5)
> 6	-	2.6 (1.2 to 5.7)^d^	-	2.6 (1.2 to 5.7)^d^
Mechanically ventilated	-	16.6 (5.9 to 46.7)^d^	-	16.6 (5.9 to 46.7)^d^
Postoperative status	-	0.2 (0.1 to 0.6)^d^	-	0.2 (0.1 to 0.6)^d^
bSCr	-	1.5 (0.9 to 2.7)	-	1.7 (1.0 to 2.9)
Documented infection	-	2.3 (1.1 to 4.8)^d^	-	2.2 (1.0 to 4.7)^d^

^a^AKI = acute kidney injury; AKIN = Acute Kidney Injury Network staging system; bSCr = baseline serum creatinine; CHUSJ = Centre Hospitalier Universitaire Ste-Justine; PICU = pediatric intensive care unit; PRISM = Pediatric Risk of Mortality. All results are presented as odds ratios (95% CI).

^b^Standard bSCr method on left side of table (or AKIN_standard bSCr _) defines bSCr as the lowest level in the three months prior to admission or normative values based on SCr for age and gender when there were no prior SCr data available. The right side of the table lists normative SCr values for age and gender used to estimate bSCr values in all patients (AKIN_all norms bSCr_) to determine the presence of AKI. ^c^Two different statistical models (or regression analyses) were performed (using stepwise backward multiple logistic regression analysis) to evaluate the independent effect of AKI on PICU mortality. In model 1, we evaluated the effect of AKI on PICU mortality, controlling only for age, gender and study site. In model 2, we evaluated the effect of AKI on PICU mortality, controlling for age, gender, study site, PRISM tertile, ventilation status, postoperative status, presence or absence of bSCr data and presence or absence of documented infection. ^d^Statistically significant odds ratios (*P *< 0.05).

**Table 3:** Association of more severe (stage 2 or 3) AKI with PICU mortality using two different approaches for determination of bSCr^a^

	Standard bSCr determinations (AKIN_standard bSCr_)^b^		Normative values for bSCr in all patients (AKIN_all norms bSCr_)^b^	
Characteristics	Model 1^c^	Model 2^c^	Model 3^c^	Model 4^c^
AKIN stage 2 or 3	9.0 (5.4 to 15.0)^d^	5.8 (3.3 to 10.4)^d^	9.9 (5.8 to 16.7)^d^	6.4 (3.6 to 11.7)^d^
Age under one year	1.8 (1.1 to 2.9)^d^	1.0 (0.6 to 1.8)	1.9 (1.1 to 3.1)^d^	1.0 (0.6 to 1.8)
Female	0.7 (0.4 to 1.1)	0.7 (0.4 to 1.3)	0.6 (0.4 to 1.0)	0.7 (0.4 to 1.2)
CHUSJ site	0.7 (0.4 to 1.1)	0.7 (0.4 to 1.3)	0.6 (0.4 to 1.0)	0.7 (0.4 to 1.2)
PRISM score, tertiles				
0 to 3 (reference group)	-	1.0 (reference)	-	1.0 (reference)
4 to 6	-	0.5 (0.2 to 1.4)	-	0.5 (0.2 to 1.5)
> 6	-	2.6 (1.2 to 5.7)^d^	-	2.7 (1.3 to 5.9)^d^
Mechanical ventilation	-	17.8 (6.3 to 50.4)^d^	-	19.2 (6.8 to 54.5)^d^
Postoperative status	-	0.3 (0.1 to 0.7)^d^	-	0.3 (0.1 to 0.7)^d^
bSCr	-	Dropped^e^	-	Dropped^e^
Documented infection	-	2.3 (1.1 to 5.0)^d^	-	2.3 (1.1 to 5.0)^d^

^a^AKI = acute kidney injury; AKIN = Acute Kidney Injury Network staging system; bSCr = baseline serum creatinine; CHUSJ = Centre Hospitalier Universitaire Ste-Justine; PICU = pediatric intensive care unit; PRISM = Pediatric Risk of Mortality; SCr = serum creatinine.

All results are presented as odds ratios (95% CI). ^b^Standard bSCr method on the left side of the table (or AKIN_standard bSCr _) lists bSCr levels as the lowest SCr level in the three months prior to admission or normative values based on SCr for age and gender when there were no prior SCr data available. The right side of the table lists normative SCr values for age and gender, which were used to estimate bSCr values in all patients (AKIN_all norms bSCr_) to determine the presence of AKI. ^c^Two different statistical models (or regression analyses) were performed (using stepwise backward multiple logistic regression analysis) to evaluate the independent effect of AKI on PICU mortality. Model 1 was used to evaluate the effect of AKI on PICU mortality, controlling only for age, gender and study site. Model 2 was used to evaluate the effect of AKI on PICU mortality, controlling for age, gender, study site, PRISM tertile, ventilation status, postoperative status, presence or absence of bSCr data and presence or absence of documented infection. ^d^Odds ratios are statistically significant (*P *< 0.05). ^e^"Dropped" means that in the backward stepwise multiple logistic regression analysis, that variable was not retained in the model and was not statistically significant.

### Acute kidney injury as a risk factor for prolonged pediatric intensive care unit length of stay and duration of mechanical ventilation

**Figure 4:**
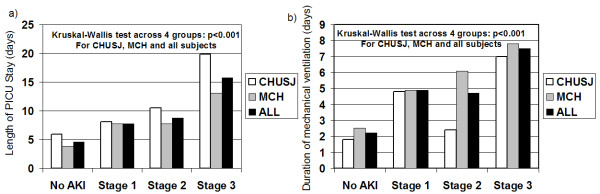
**Association of PICU length of stay and mechanical ventilation duration with increasing AKI severity**. Histogram illustrating **(a) **duration (in days) of pediatric intensive care unit (PICU) stay and **(b) **duration (in days) of mechanical ventilation in patients with no acute kidney injury (AKI) or Acute Kidney Injury Network (AKIN) stage 1 to 3 AKI. White bars represent Centre Hospitalier Universitaire Ste-Justine (CHUSJ) data, gray bars represent Montreal Children's Hospital (MCH) data and black bars represent all data. A nonparametric Kruskal-Wallis test was used to evaluate significant differences across the four AKI severity strata.

Patients with AKI had approximately double the duration of PICU LOS and of mechanical ventilation compared to non-AKI patients (Table 1). Figure 4 displays graphically how PICU LOS and duration of mechanical ventilation increased in stepwise fashion with worsening AKI status at both study sites. In multiple linear regression analyses, the presence of any AKI and AKIN stage 2 or worse were persistently associated with longer PICU LOS and longer duration of mechanical ventilation, independent of other variables and regardless of which bSCr measurement method was used to determine AKI status (*P *< 0.005 in all analyses). When patients who died in the PICU were excluded, the results were almost identical (data not shown). We also performed these analyses excluding (1) children younger than three months of age (*n *= 333) and (2) children younger than one month of age (*n *= 226), since there are few studies validating the use of the AKIN staging system in neonates. The results regarding AKI-outcome associations were essentially identical in terms of both magnitude and significance (not shown).

### Predicting acute kidney development on the basis of early pediatric intensive care unit serum creatinine increases

**Figure 5:**
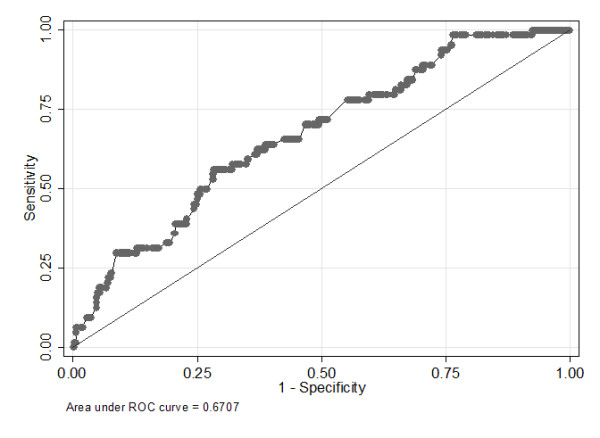
**Predicting AKI using PICU admission day 1 percentage SCr increase in patients without admission AKI**. Area under the receiver operating characteristic curve (ROC) demonstrating the ability of first pediatric intensive care unit (PICU) admission day of percentage serum creatinine (SCr) increase from baseline to predict future acute kidney injury (AKI) development after PICU admission day 1. This analysis includes only patients with no AKI on PICU admission day 1 who had their SCr levels measured on PICU admission day 1. Only patients from the Montreal Children's Hospital are included in this analysis.

In 800 patients admitted to the MCH PICU who did not already have AKI on admission day 1 and who had SCr measured on admission day 1, we evaluated the diagnostic characteristics of admission day 1 percentage SCr increases to predict the development of AKI on admission day 2 or later. In other words, we evaluated early SCr increase (< 50%) as an early biomarker of AKI (≥ 50% SCr increase). The receiver operating characteristic curve of day 1 percentage SCr increase to predict future AKI is displayed in Figure 5. The AUC was 0.67 (95% confidence interval 0.60 to 0.74). A day 1 percentage SCr increase from a baseline cutoff of 25% (using the traditional bSCr method) predicted future AKI with specificity of 81% and sensitivity of 33%.

## Discussion

We have validated findings from previous single-center reports that AKI is common and independently associated with poor PICU outcomes. In addition, we evaluated two approaches to assessing bSCr which are likely to be used for either prospective or retrospective database studies and found that, regardless of the measurement method used, AKI-outcome associations persist. We also found that in a substantial proportion of children, SCr level does not return to baseline by the time of PICU discharge.

Our finding that AKI occurs extremely early after PICU admission validates findings of other single-center studies [5,8,12]. The implication of this finding is of paramount importance. A voluminous amount of research has been devoted to early AKI detection by using novel biomarkers of AKI [17-21], since SCr is thought to increase late with AKI and, for AKI treatments to be successful, early biomarker application is necessary [22,23]. However, applying these new biomarkers to the PICU population will clearly be a great challenge if most children already have AKI within 24 to 48 hours of PICU admission. Future PICU AKI clinical trials may need to apply new AKI biomarkers even prior to ICU admission. In a previous study of children undergoing cardiac surgery, we found that early increases in SCr (< 50%) moderately predicted future overt AKI [4]. In the analyses presented in this article, we found that in the subgroup of children who did not have AKI at the time of PICU admission, the percentage increase in SCr had moderate predictive value for determining who would or would not develop AKI in subsequent days (AUC = 0.67). Specifically, a 25% SCr increase predicted future AKI with specificity of 81% but sensitivity of only 33%. Therefore, the presence of a 25% increase may be useful for predicting future AKI development, but lack of a 25% increase does not provide much information. This simple and inexpensive way of predicting AKI events should also be examined in adults.

There is almost no information available on what happens to children with AKI after the AKI event occurs. Yet, in adults, it is known that AKI is a significant predictor of postdischarge mortality and chronic kidney disease [24,25]. As a first step toward understanding the natural history of PICU AKI, we have shown that SCr does not return to bSCr values at the last PICU SCr assessment in many children who develop AKI and that those with worse AKI are less likely to return to bSCr values. This finding has several potential implications. First, it is possible that children whose SCr levels do not return to bSCr concentrations may be those at highest risk for long-term renal abnormalities. This should be confirmed in prospective studies, and our results provide a starting point for determining the at-risk population that might be studied. Second, this finding may also demonstrate a problem in the process of care which requires future study. Anecdotally, children who leave the PICU with "normal" SCr levels likely do not have follow-up examinations of renal function. However, clearly, a substantial portion of children do not leave the PICU with "normal renal function" compared to bSCr levels.

We evaluated two approaches to estimating bSCr. This follows from our previous research demonstrating that, depending on which bSCr measurement is used, AKI patterns, incidence and outcome associations may differ substantially [14]. However, bSCr is often unknown in children admitted to the PICU; therefore, applying the "true" bSCr to determine AKI status is extremely challenging. Moreover, in future studies, which may be based on provincial or state databases wherein prehospitalization SCr data may not be readily available, it is extremely worthwhile to understand the extent to which alternative methods of estimating bSCr may affect study findings. As shown in the current study, bSCr was unknown in over 50% of the study population. On the basis of our previous research, we evaluated what we felt was the most reasonable and reproducible method of estimating bSCr, which was to use center-specific age- and gender-based normative values in all patients versus only in those with an unknown bSCr. Particularly in children, this is reasonable, given that the prevalence of chronic kidney disease is extremely low. Despite finding evidence of differences in AKIN classification when comparing both bSCr methods, regardless of the bSCr method used, the AKI-outcome associations were maintained. This suggests that using normative estimated bSCr may be a reasonable alternative when "true" bSCr data are unavailable, which is particularly likely to occur in population-based studies.

One of the biggest strengths of our study is that the data were from two different university health care centers. Although both centers are located in the same city, practice patterns and processes of care differ between them, thus increasing the generalizability of our findings and enhancing our validation of findings from previous studies. We were careful to perform our statistical analyses in a targeted fashion in an attempt to study the most parsimonious statistical models and avoid overfitting of the data. This is the first AKI study in pediatric patients to evaluate the return of SCr to baseline values after AKI and the first to explore how early SCr increases may aid in predicting AKI in this population. In addition, it is important to note that the PRISM score does not include an assessment of renal status, thereby alleviating concerns regarding colinearity of AKI and illness severity score in the statistical models. However, the physiologic relationship between AKI and mechanical ventilation is complex, and colinearity between these two elements of the statistical models cannot be completely ruled out. An inherent limitation of this study is its partial retrospective nature. For example, we were unable to include one of the criteria of the AKIN definition for ascertaining AKI status: that the increase in SCr should occur over a period of 48 hours. A positive aspect of this criterion is that it leads to less misclassification of patients who develop a slow SCr increase throughout admission as having AKI. However, in the case of children, almost all AKI occurs within the first few days after PICU admission (Figure 3); therefore, it is unlikely that a substantial proportion of patients with a slow, prolonged increase in SCr were misclassified as having "true" AKI. It will be worthwhile in future studies to specifically evaluate the extent to which this "48-hour increase" criterion leads to differences in AKI diagnoses and study conclusions in pediatric populations, particularly since this criterion is often difficult to ascertain. Another limitation is that because we sought to combine only those variables from both databases which we determined to have been collected in a similar fashion, we were unable to assess other potentially important factors such as the use of nephrotoxic medication or specific diagnoses (for example, sepsis or hematologic and oncologic diseases). Some of our analyses were limited to the MCH center because data were unavailable at the CHUSJ center. Finally, it is important to note that we were unable to evaluate the pRIFLE criteria for AKI in comparison to the AKIN staging system, since we did not have complete data regarding patients' height. Particularly in small children, in whom small changes in SCr may have a large effect on AKI classification, it is possible that conclusions regarding AKI-outcome associations or the effect of age as an AKI risk factor might differ when using the pRIFLE criteria. When we excluded neonates from the analyses evaluating AKI as a predictor of PICU LOS and duration of mechanical ventilation, our results were unchanged. However, in future studies, investigators should strongly consider determining to the extent to which pRIFLE versus AKIN staging may lead to different conclusions, especially in young children.

## Conclusions

AKI is an important risk factor for poor outcomes in critically ill children. Future studies should elucidate the effects of AKI on long-term renal and nonrenal outcomes. Clinical trials of AKI are needed, but challenges will be associated with preventing PICU AKI because of early occurrence during admission. While SCr is an imperfect AKI biomarker, early small SCr increases may provide an important clue that critically ill children will develop overt AKI and be at risk for negative effects of AKI, such as fluid overload and medication toxicity.

## Key messages

• AKI is common in critically ill children and is associated with increased hospital mortality and morbidity.

• AKI in children occurs very early during PICU admission, which has significant implications for the conduct of future pediatric AKI clinical trials and biomarker studies of AKI.

• The use of age- and gender-based normative values for estimating bSCr or reference SCr is reasonable when evaluating the effect of AKI on outcomes in pediatric patients.

• Small acute SCr increases (< 50% from bSCr) may be a cost-effective and simple method to predict the future occurrence of more significant AKI.

• About one-fifth of children leave the PICU with abnormal SCr levels.

## Abbreviations

AKI: acute kidney injury; AKIN: Acute Kidney Injury Network; AUC: area under the curve; bSCr: baseline serum creatinine; CHUSJ: Centre Hospitalier Universitaire Ste-Justine; LOS: length of stay; MCH: Montreal Children's Hospital; PICU: pediatric intensive care unit; PICUE: pediatric intensive care unit evaluation; pRIFLE: Pediatric Risk, Injury, Failure, Loss, End-Stage Renal Disease criteria; PRISM: Pediatric Risk of Mortality; SCr: serum creatinine; SD: standard deviation.

## Competing interests

The authors declare that they have no competing interests.

## Authors' contributions

OA was involved in the study design, data collection and the writing of several portions of the manuscript. KAE was involved in the study design, data collection, interpretation of results and the writing of several portions of the manuscript. AH performed a majority of the database merging and performed statistical analyses and generation of variables. FG coordinated the data collection pertinent to this study, reviewed relevant variables for merging and participated actively in all aspects from data analysis to final manuscript preparation. TD made decisions regarding variable merging between databases (conceptual and technical), performed extraction of relevant variables and was actively involved in the statistical approach and the interpretation of the results. RG was responsible for the PICUes database and was involved in the study design phase, the interpretation of results and the writing of the manuscript. VP was actively involved in the data/variable-merging decision-making phase regarding renal variables as well as in the interpretation of results and discussions. MZ was involved in all aspects of the study, including designing the study, data collection, database management, the majority of the statistical analyses and the writing of the manuscript. All authors read and approved the manuscript submitted for publication.
